# Perioperative management of Takotsubo cardiomyopathy: an overview

**DOI:** 10.1186/s44158-024-00178-y

**Published:** 2024-07-15

**Authors:** Marta Pillitteri, Etrusca Brogi, Chiara Piagnani, Giuseppe Bozzetti, Francesco Forfori

**Affiliations:** 1 Department of Anaesthesia and Intensive Care, University of Pisa, Pisa, Italy; 2 Neuroscience Intensive Care Unit, ASST Grande Ospedale Metropolitano Niguarda, Piazza Ospedale Maggiore, 3, 20162 Milan, Italy; 3 Department of Anaesthesia, Peri Operative Medicine and Critical Care, NHS Golden Jubilee, Glasgow, UK

**Keywords:** Takotsubo cardiomyopathy, Takotsubo syndrome, Broken heart syndrome, Left ventricular apical ballooning syndrome, Stress cardiomyopathy, Perioperative period, General anaesthesia

## Abstract

Resembling the morphology of Japanese polyp vessels, the classic form of Takotsubo cardiomyopathy is characterized by the presence of systolic dysfunction of the mid-apical portion of the left ventricle associated with basal hyperkinesia. It is believed that this may be due to a higher density of β-adrenergic receptors in the context of the apical myocardium, which could explain the greater sensitivity of the apex to fluctuations in catecholamine levels.

The syndrome is precipitated by significant emotional stress or acute severe pathologies, and it is increasingly diagnosed during the perioperative period. Indeed, surgery, induction of general anaesthesia and critical illness represent potential harmful trigger of stress cardiomyopathy. No universally accepted guidelines are currently available, and, generally, the treatment of TTS relies on health care personal experience and/or local practice. In our daily practice, anaesthesiologists can be asked to manage patients with the diagnosis of new-onset Takotsubo before elective surgery or an emergent surgery in a patient with a concomitant stress cardiomyopathy. Even more, stress cardiomyopathy can arise as a complication during the operation.

In this paper, we aim to provide an overview of Takotsubo syndrome and to discuss how to manage Takotsubo during surgery and in anaesthesiologic special settings.

**Supplementary Information:**

The online version contains supplementary material available at 10.1186/s44158-024-00178-y.

## Introduction

Takotsubo syndrome (TTS) is an acute myocardial syndrome characterized by a peculiar, transient abnormal cardiac wall motility (apical ballooning), without impairment of coronary arteries, which can cause severe cardiac failure [1]. The syndrome is precipitated typically by significant emotional stress or acute severe pathologies (e.g. acute subarachnoid haemorrhage) accompanied by an increased activation of the sympathetic nervous system [1, 2]. Symptoms mimic the myocardial infarction, and the clinical manifestation can be various such as chest pain, ECG abnormalities, increase in serum troponins and heart failure [2, 3]. Mortality can reach 4–5%, secondary to cardiac arrest and heart failure. Other cardiac complications may occur as well [2].

TTS is classified as primary (the one following an emotional stress), and secondary, when TTS arise after physical stress or trauma [3]. Generally, the primary form affects postmenopausal women, while there are no preferences in gender or age in the secondary form [2, 3]. Examples of physical triggers of secondary TTS include, but are not limited to, stroke, cerebral bleeding, pneumonia, bronchitis, surgery, gastrointestinal bleeding, pheochromocytoma, giving birth, cancer, sepsis and bone fracture [4]. Consequently, Takotsubo may occur during the perioperative period as well as in the intensive care unit [5]. Indeed, surgery, induction of general anaesthesia and critical illness all represent potential harmful triggers for the transient left ventricular apical ballooning syndrome [6]. The excessive release of catecholamine leads to myocardial stunning with regional ballooning patters [7]. Typically, the apical ballooning patter is observed; however, an increasing number of atypical form cases (e.g. midventricular ballooning syndrome) were described in the last few years [8]. Therefore, TTS should be considered in the differential diagnosis of left ventricular dysfunction or ECG abnormalities suggesting acute myocardial infarction in perioperative setting [5].

Due to the increasing awareness of the possibility of managing a patient who suddenly develops TTS in our daily anaesthesiologic practice, and the lack of clear treatment guideline, we provide an overview of TTS syndrome and how to manage Takotsubo during surgery. Some anaesthesiologic special settings are also highlighted.

## Physiopathology

**Fig. 1:**
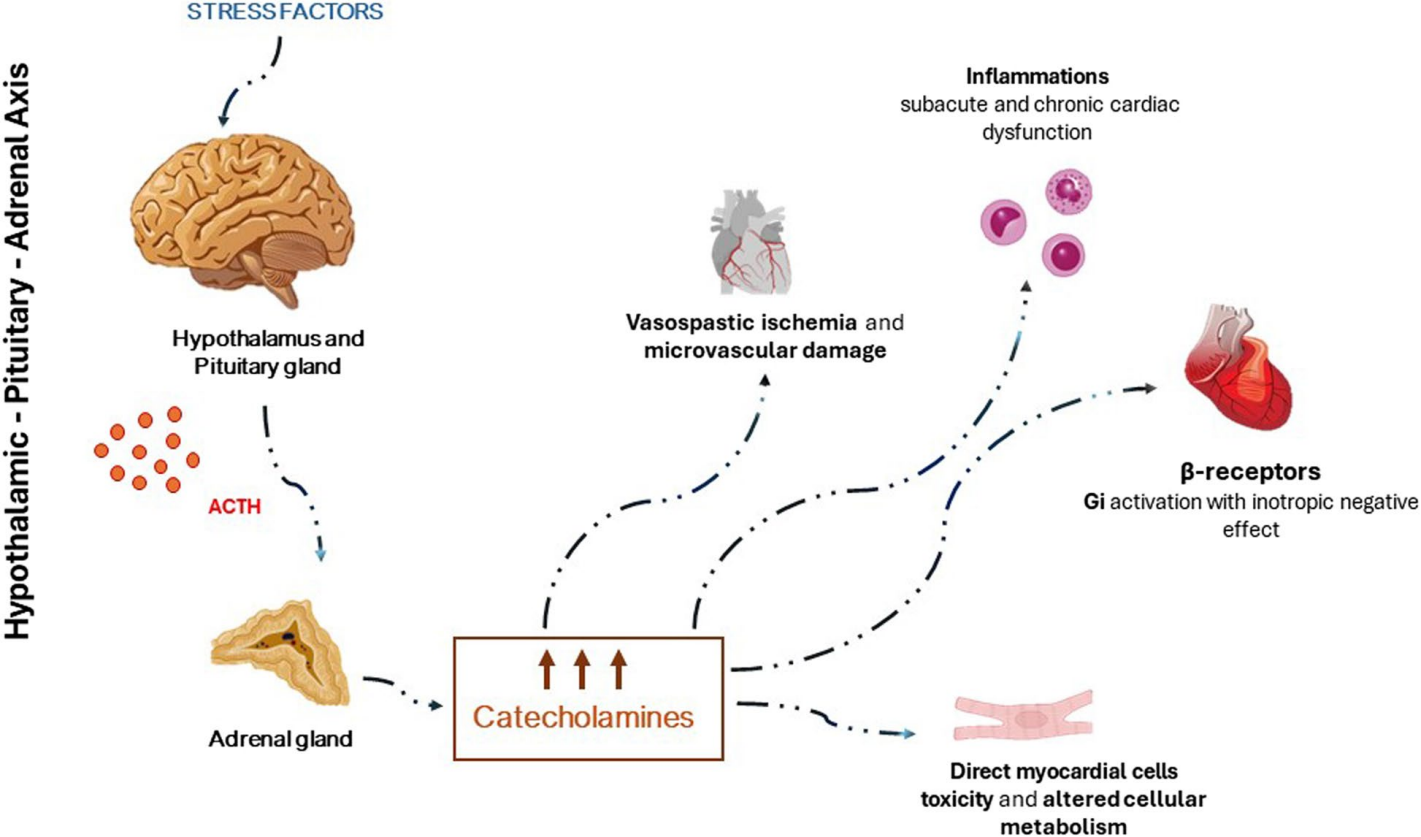
Pathophysiological of Takotsubo syndrome

The exact pathophysiology of TTS is unknown; however, an acute adrenergic hyper stimulation is suspected as the primary cause (Fig. 1). Up to now, the excessive catecholamine release is considered the most widely conventional pathophysiologic mechanism of Takotsubo [9]. The first correlation between TTS and excessive catecholamine release was described in a patient with pheochromocytoma [10, 11]; further observational studies and case reports evaluating the concentration of catecholamines in patient with Takotsubo confirmed this observation [12, 13].

The prompt adaptative response to a severe stress leads to the activation of a specific neural network. The brain cortex, the amygdala and the limbic system represent essential areas responsible for the recognition and the activation of these adaptative neural networks with the consequent activation of the brain stream and adrenomedullary pathways [14]. The hypothalamic–pituitary–adrenal axis has been considered the main responsible of the overproduction of catecholamines after its activation by norepinephrine produced by locus coeruleus, secondary to a strong emotional or physical stress [10, 15]. Even more, the direct release of catecholamines locally at myocardial level may also occur by cardiac sympathetic nerve terminals activated by the descending neural pathway from the rostral pons [10, 16].

If the high level of catecholamines has been considered a trigger for the development of TTS, the response in myocardial contractility also plays an important role. The variable density of β-1 and β-2 receptors on the heart has been suggested as a possible explanation of the typical Takotsubo segmental abnormalities [17]. β adrenergic are G protein-coupled receptors and regulate several physiological cell function and the excitation–contraction coupling of myocardium [18]. In the heart, the majority of β receptors are subtype 1, which induce chronotropic and inotropic positive effects, due to the G_s_-adenylyl cyclase (AC)-cAMP-protein kinase A (PKA) signalling cascade [19]. On the contrary, β-2 receptors are more distributed in the cardiac apex. Even more, β-2 receptors couple both Gs proteins and Gi (G inhibitory) proteins. The activation of Gi has negative effects on cardiac contraction. Noteworthy, in the presence of an excess of adrenergic stimulation, there is a switch of G proteins resulting in a prevalence of Gi that leads to an inotropic negative effect and potential left ventricular dysfunction [20]. The distribution of β receptors subtype 2 can explain the typical apical ballooning pattern [13], even though it is a simplification of the more complex physiology as the adrenergic intracellular signalling is interconnected [21]. While the pathophysiological hypothesis based on β-2 receptor distribution well describes the typical Takotsubo syndrome, it is incomplete for the interpretation of the pathophysiology of atypical Takotsubo with impaired basal wall motion. A hypothesis could be the overactivation of β-2 receptors at the heart base without the simultaneous activation of them in the apex. Interestingly, a “wandering” type of Takotsubo was also observed, characterized by an atypical evolution from apical to mid ventricular ballooning motion [22]. Differences in contractile patterns can be also attributed to the variation of the structural organization and molecular differences in adrenoceptor signalling between cellular membranes of cardiomyocytes in the apex and in the basal [23].

Even more, high concentrations of catecholamines have other severe adverse effects. Catecholamines can cause direct myocardial cell toxicity damage by a sudden influx and impaired clearance of intracellular calcium which results in prolonged contraction and ATP depletion, an effect termed “catecholamine toxicity” [24]. Calcium overload, oxidative stress and mitochondrial dysfunction are all possible mechanisms implicated in the cardiotoxicity, leading to myocardial injury and cellular necrosis [25]. Furthermore, the increased metabolic demand, due to increased heart rate and cardiac contraction, and the reduced blood and oxygen supply, due to vasoconstriction and vasospasm, further raise the risk of ischemic events and cell death [26]. The histological studies conducted in the experimental model of TTS showed typical contraction band necrosis with mononuclear cell infiltration, damaged mitochondria, lipid drops and oedema [27]. In addition, the prolonged exposure of the heart to high levels of catecholamines can induce vasospastic ischaemia which worsen the cardiac injury. Several studies in the past few years tried to investigate whether coronary or epicardial vascular ischaemia could have a role in the pathogenesis of TTS; however, no signs of coronary ischaemia have been demonstrated during invasive exams (e.g. coronarography) in the acute phase of TTS, while signs of epicardial hypoperfusion have been attributed to altered myocardial motion and diastolic dysfunction [2, 28]. In addition, inflammation also occurs, as a direct consequence of microvascular damage and stunned myocardium. The inflammatory response might contribute to the subacute and chronic cardiac dysfunction after TTS, especially, when no recovery is seen [2]. An overview of passible physiopathological mechanisms is shown in Fig. 1.

## Clinical presentation and diagnosis

As already stated, Takotsubo syndrome lacks distinctive elements in the clinical presentation that can make the diagnosis immediately unequivocal. Historically, the emotional triggers associated with stress cardiomyopathy were considered primarily negative, hence the name “Broken Heart Syndrome”. More recently, however, a subgroup of patients, presenting with stress cardiomyopathy following an intense, but positive, emotion, was described. Since the clinical presentation of these forms also differed from the classic one, being more frequent in men and showing atypical ballooning patterns (most often midventricular), the new definition of Happy Heart Syndrome was introduced [29]. However, in about a third of cases, it is not possible to clearly trace a triggering event, a factor which contributes to making the diagnosis less immediate.

The clinical manifestations, the electrocardiographic changes together with the variable levels of cardiac enzymes and the hemodynamic perturbations during stress cardiomyopathy are indistinguishable from those of the classically known acute coronary syndromes: STEMI, N-STEMI and unstable angina. The most prevalent symptom, according to data from the International Takotsubo registry, is anginal-type retrosternal chest pain. Often, in patients diagnosed with Takotsubo, the onset of pain follows an emotional or physical stress of various kinds [30]. Chest pain is frequently accompanied by other symptoms such as dyspnoea and syncope, but arrhythmias, electrocardiographic changes and general clinical signs of heart failure may also be present [31, 32]. In a minority of cases, the onset can occur with a picture of full-blown cardiogenic shock.

More precisely, we distinguish primary forms of stress cardiomyopathy: generally outpatients that more often have chest pain and dyspnoea as presenting symptoms, from secondary forms, which typically affect patients already hospitalized for other critical pathologies and usually manifest themselves with signs and symptoms of acute heart failure, such as pulmonary oedema, arrhythmias or cardiac arrest [4]. The Anaesthetist and Critical Care specialist must have a high degree of suspicion for both forms. Indeed, if, on the one hand, the secondary form can be insidious to identify in a clinically complex patient, on the other hand, stress cardiomyopathy can arise suddenly even in an otherwise healthy patient hospitalized for elective surgery, representing a totally unexpected complication [5]. The incidence of Takotsubo in the perioperative period seems to be approximately one case every 6700 surgical interventions [33].

**Fig. 2:**
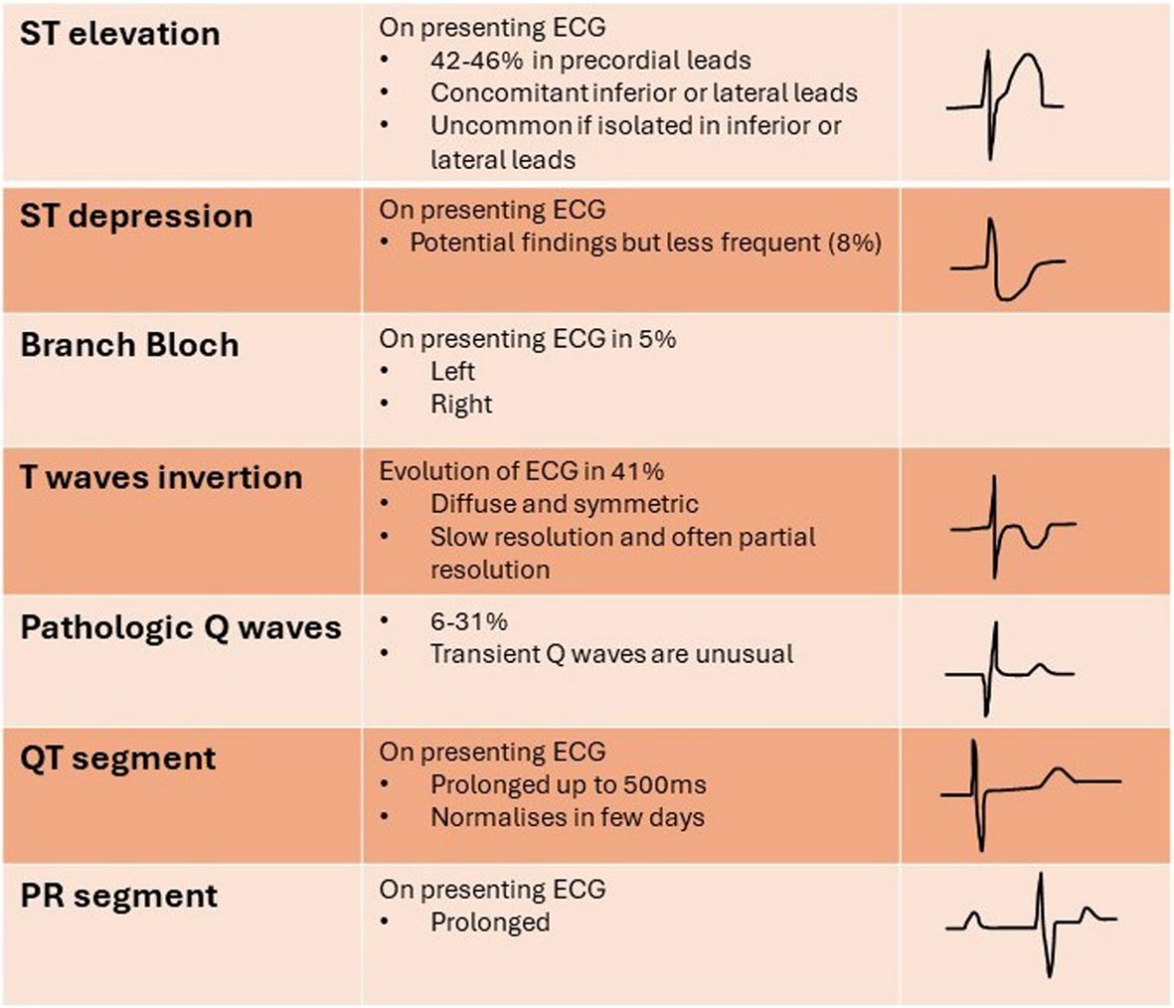
Possible electrocardiographic alterations observed in Takotsubo syndrome

The electrocardiographic changes that are most typically seen during stress cardiomyopathy include, but are not limited to, ST-segment elevation, T-wave inversion, or ST-segment changes and QT prolongation [34]. These alterations are non-specific. The ECG patter is influenced by anatomical location of the ventricular ballooning. ST elevation is generally observed in precordial lateral and apical ECG leads; typically, inversed aVR is an associated feature. More precisely, ST elevation can be generally spotted in precordial V2-V5 leads and Leads II [35]. As already stated, these alterations are non-specific, and coronarography is mandatory for the differential diagnosis with ACS. Progressive T-wave inversion is also common in TTS, generally characterized by symmetric and diffuse distribution with slow and often partial resolution [36]. An overview of possible electrocardiographic patterns is presented in Fig. 2.

Troponin blood level during Takotsubo is generally lower than the values observed during STEMI, but these values are comparable to those observed during NSTEMI. A similar trend is observed for CK and CK-MB [37, 38]. BNP levels are generally greater than that observed in acute coronary syndromes, and a high BNP/troponin index can generally direct suspicions towards the former. A study by Doyen et al. highlighted that a ratio greater than 159 has high sensitivity and specificity (95.2% and 97.9%, respectively) for distinguishing Takotsubo from myocardial infarction [39]. However, serological tests alone are not sufficient to distinguish stress cardiomyopathy from acute coronary syndromes, and other clinical findings have to be taken into account.

The diagnosis of stress cardiomyopathy is based on the demonstration of a reversible regional motility abnormality of the left ventricular wall that extends beyond the territory perfused by a single coronary vessel [35]. This can be observed by performing coronary arteriography, left ventriculography and echocardiography. As suggested by the name, referring to the morphology of Japanese polyp vessels, the classic form of Takotsubo cardiomyopathy is characterized by the presence of systolic dysfunction of the mid-apical portion of the left ventricle associated with basal hyperkinesia.

**Fig. 3:**
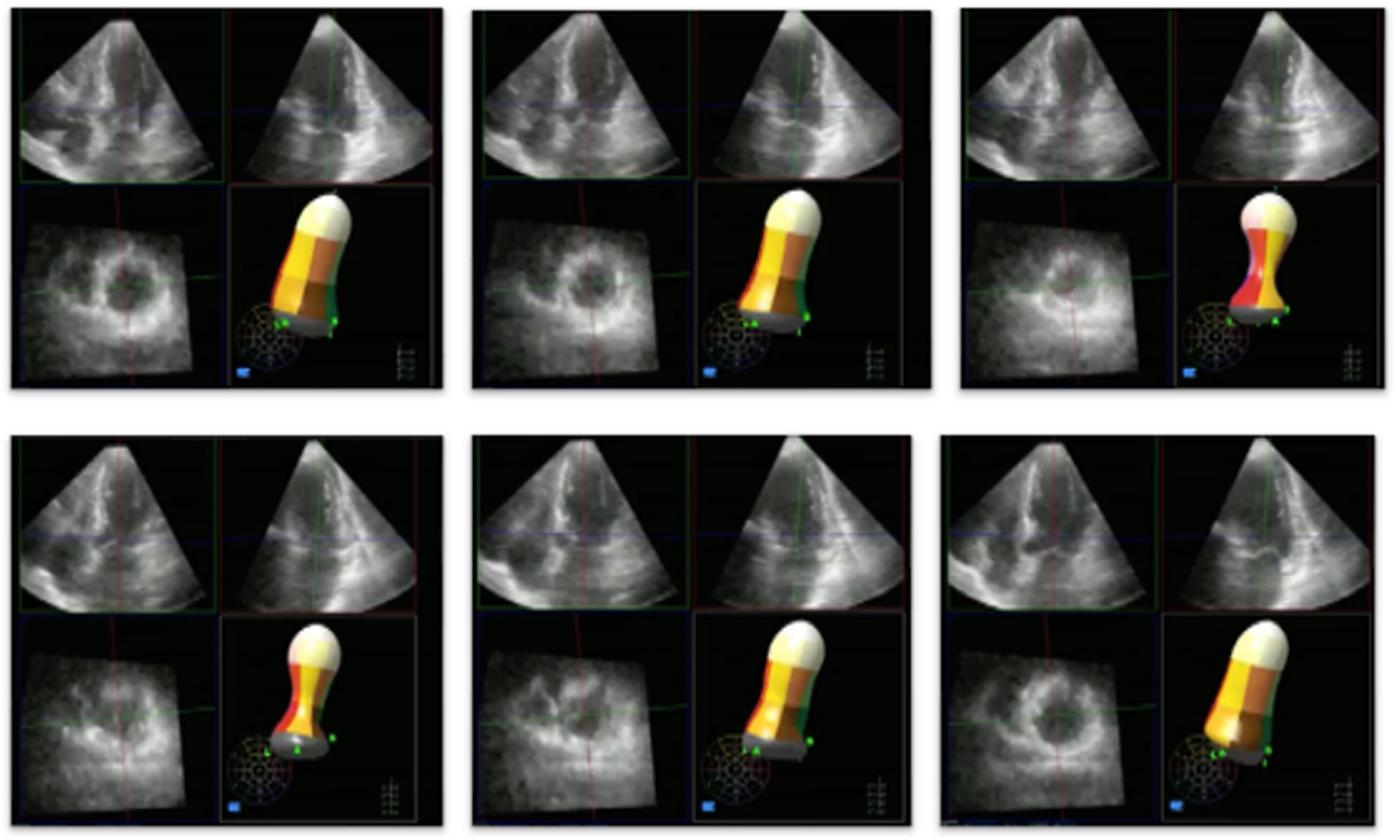
Subsequential echocardiographic 3D images of ejection fraction of a typical form of TTS

**Fig. 4:**
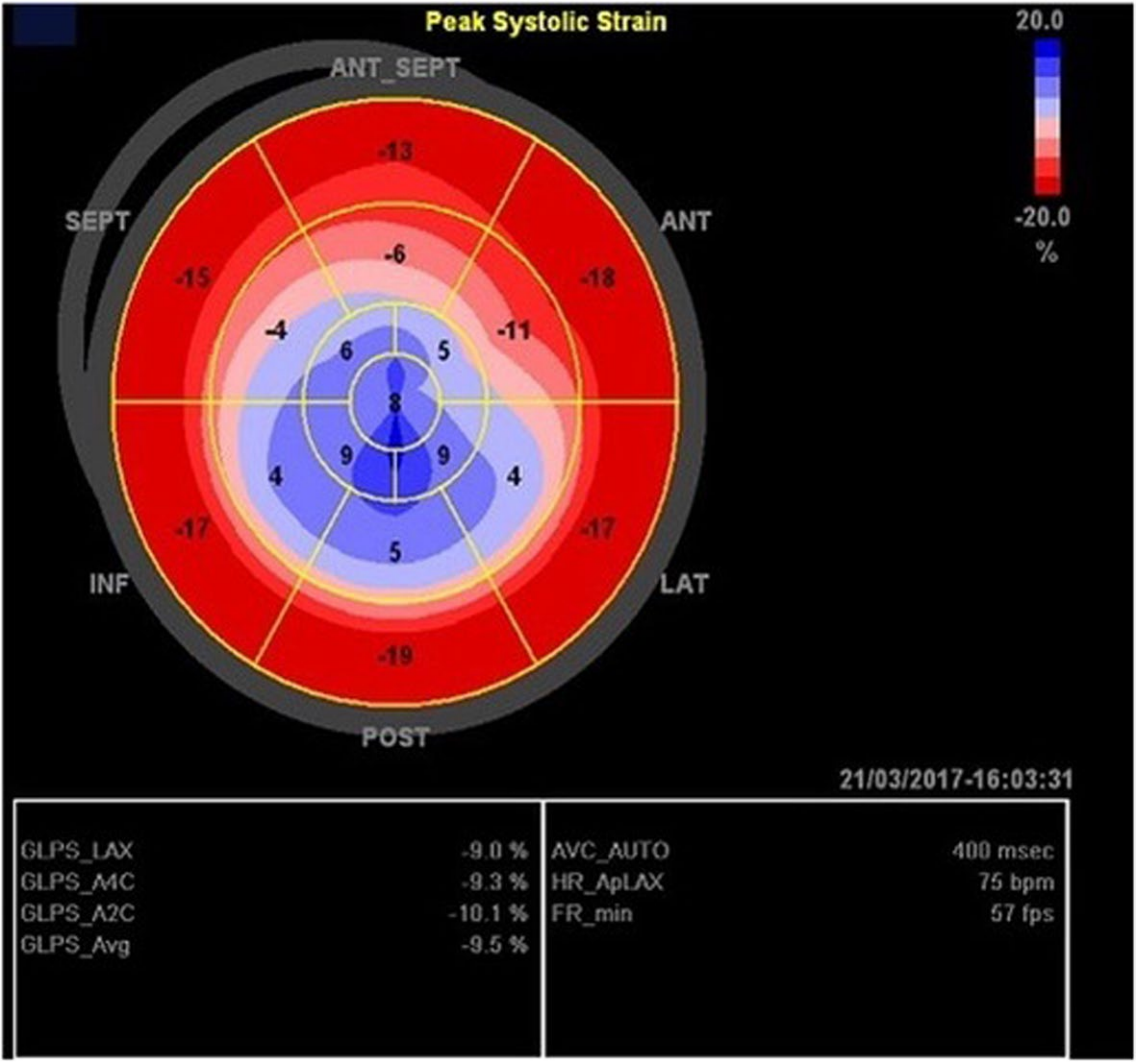
Systolic longitudinal strain pattern of a typical form of TTS: total lack of shortening of the myocardial apical segments (blue colour), indicating loss of contractility, and normal shortening pattern of the basal areas (red colour)

Transthoracic echocardiography usually is the first imaging choice, and it is very helpful to identify the morphological and functional characteristics of TTS. In the typical form, which accounts for most of the cases, TTS displays wall motion abnormalities (akinesia or dyskinesia) involving the apical segments (“apical ballooning”) and, to some extent, the midventricular region (Figs. 3 and 4 and Additional file 1: Supplemental material 1). The basal segments are generally hyperkinetic. The global ejection fraction is depressed in the acute phase and usually recovers after approximately 2 weeks [40]. The wall motion abnormalities (WMAs) of TTS involve the myocardial segment in a very symmetrical way. This circumferential pattern differs from ischemic-induced WMAs, in which the primary contractility deficit is visible mainly where the culprit coronary lesion is. However, the rate of recovery differs among the myocardial regions, with the anterolateral segments seemingly recovering earlier [41].

The WMAs and the global dysfunction can be very well visualized with the 3D technique. Alongside the classic apical ballooning TTS, here are some variants that have been described: the “midventricular” and “inverted”. The midventricular is characterized by akinesis of the midventricular segments, hypercontractility of the base and mild hypokinesis or normal contraction of the apical segments. The inverted form can be divided into the “apical sparing” variant, where the contractility is well preserved in the apical regions with severe hypokinesis of the remaining segments and the “reverse” variant, where the hypokinesia involves just the basal segments.

Echocardiography can also identify the presence of diastolic dysfunction (DD), often present in the acute phase. The DD can be displayed by conventional techniques (transmittal flow, tissue Doppler), according to guidelines, or more advanced ones (left atrial strain analysis).

The myocardial deformation imaging (speckle tracking strain) can also be applied to the global ventricular function: the longitudinal, radial and circumferential strain are all impaired in the myocardial regions involved. Interestingly, the global LV twist and untwisting are also impaired. Just like the WMAs and LV EF, the deformation parameters are all reversible. While assessing TTS with echocardiography, it is important to rule out the presence of LVOT obstruction, defined as an intraventricular gradient of > 25 mmHg). This complication has obvious therapeutic implications and can occur in the typical TTS form. The LVOT obstruction can be associated with systolic anterior motion (SAM) of the mitral valve and related mitral regurgitation (MR). However, the MR in TTS can have other causes (papillary muscle displacement and tethering, particularly when the EF is severely depressed). It is therefore important to carefully assess the anatomy of the mitral valve, sometimes with the transoesophageal approach if the transthoracic images are not clear enough.

It is also imperative to rule out thrombi in the LV apex, particularly in the typical TTS form, usually occurring in the first 2 days after the onset of the cardiomyopathy. The thrombi can be mural or pedunculated. In around 15% of the cases, the right ventricle (RV) can also be involved: similarly to the LV, the RV apex is typically akinetic with hyperkinesia of the basal segments. This aspect has been described as “reverse McConnel sign”[42]. In those cases, the RV dysfunction can further help in differentiating TTS from ischemic forms.

Various diagnostic criteria have been proposed over the years [31, 43]. However, with the growing awareness of Takotsubo as a clinical entity in its own right, new diagnostic criteria have been formulated by the Heart Failure Association of the European Society of Cardiology, responding to the need for the inclusion of atypical and secondary forms that previously were not clearly defined [1]. Furthermore, the attempt to adopt a more pragmatic approach resulted in the formulation by the expert committee of the Takotsubo Registry of a probability score (Intertak) in patients presenting with chest pain and/or dyspnoea. This score combines five variables in the medical history and two electrocardiographic variables [34, 44] (as shown in Table 1). The score facilitates the distinction of stress cardiomyopathy from an acute coronary syndrome, preventing some patients who present without ST-segment elevation on the electrocardiogram from undergoing coronary angiography or other unnecessary invasive diagnostic investigations.

**Table 1:** International Takotsubo (InterTAK) diagnostic score. The maximal score yields 100 points

Variable	Maximum points
Female sex	25
Emotional stress	24
Physical stress	13
No ST-segment depression	12
Acute, former or chronic psychiatric disorder	11
Acute, former or chronic neurologic disorder	9
Prolonged QTc time (Female > 460 ms; male > 440 ms)	6

## Complications

Stress cardiomyopathy is generally a transient condition, with most patients recovering the systolic ventricular function within weeks after the acute event [45, 46]. However, the risk of complications during the acute phase is not negligible, and these can be further aggravated by the underlying clinical situation (e.g. respiratory, renal insufficiency, sepsis, subarachnoid haemorrhage). Indeed, cardiogenic shock can occur in up to 20% of the cases, especially in the biventricular and isolated forms of the right ventricle and it represents an important cause of mortality during the acute phase [47]. The development of shock is surely an expression of the degree of left ventricular dysfunction; however, left ventricles outflow tract obstruction and possible mitral regurgitation can be contributing factors of haemodynamic instability.

Left ventricular outflow tract obstruction occurs in approximately 20% of patients with stress cardiomyopathy and is characteristically associated mainly with the typical forms with apical or midventricular ballooning and basal hyperkinesia; in 10–20% of cases, a certain degree of mitral regurgitation can be added due to anterior systolic movement of the valve apparatus [47, 48]. Generally, LVOTO is a phenomenon that occurs in hypertrophic cardiomyopathies, in which some characteristic structural changes, including the asymmetric hypertrophy of the interventricular septum or, more generally, the increased thickness of the ventricular walls can lead to a state of narrowing of the left ventricular cavity, particularly in the outflow tract [49]. Due to this phenomenon, the Venturi effect caused by the increased velocity of blood in the outflow tract attracts the mitral valve in an anterior direction. This phenomenon is particularly accentuated in those patients with structural abnormalities of the valve itself, including, for example, floppy and elongated valve leaflets, or other sub valvular apparatus anomalies [50]. The anterior movement of the mitral valve during systole essentially has two main consequences: on one hand, the mechanical obstruction of the outflow tract, which reduces the ejection of blood into the aorta during systole; on the other hand, the mitral insufficiency that further reduces the forward stroke volume and can cause congestion in the left atrium contributing to the onset of pulmonary oedema. The factors that worsen LVOTO are all those that cause increased flow velocity and reduction of lumen at the end of the systole in the outflow tract, and therefore an increase in the gradient. Positive chronotropic and inotropic drugs, states of augmented endogenous production of catecholamines and conditions of reduced preload and afterload can trigger an LVOTO that would be insignificant under basal conditions. In typical Takotsubo, septal hypokinesia alters septal angulation and this, associated with baseline hyperkinesia and possible myocardial oedema, contributes to LVOTO. Therefore, LVOTO is generally a complication of the acute phase of Takotsubo, although forms of LVOTO with a so-called late onset, (i.e. after the resolution of wall motility abnormalities) have been described. Nevertheless, the etiopathogenesis of these forms is still largely unknown, and pre-existing forms of myocardiopathy probably play a role [51].

Even if malignant arrhythmias are reported as less frequent in TTS in comparison to STEMI, an increased occurrence of atrial or ventricular arrhythmias may be observed in patients with stress cardiomyopathy [52]. Atrial fibrillation is by far the most frequent arrhythmia, with a reported prevalence of up to 25%, and appears to be associated with increased long-term mortality [53]. Ventricular arrhythmias are rarer than atrial ones but have been described in 5–9% of cases in the acute phase. Among the most observed, a torsade de pointes can arise in the context of a lengthening of the QT interval [54, 55]. The formation of mural and intracardiac thrombosis represents an additional severe complication of TTS. Blood stasis in the akinetic portions of the ventricle can favour the formation of mural thrombi; these may likely represent the origin of systemic embolisms found in the course of stress cardiomyopathy with possible pathophysiological explanation of ischaemic stroke observed in these conditions [56, 57]. Finally, intramyocardial haemorrhage and rupture of the free wall of the ventricle have also been reported in rare cases, usually occurring at sites of extensive ischaemia–reperfusion injury. They are generally associated with poor outcome [58].

## Takotsubo during perioperative period

**Fig. 5:**
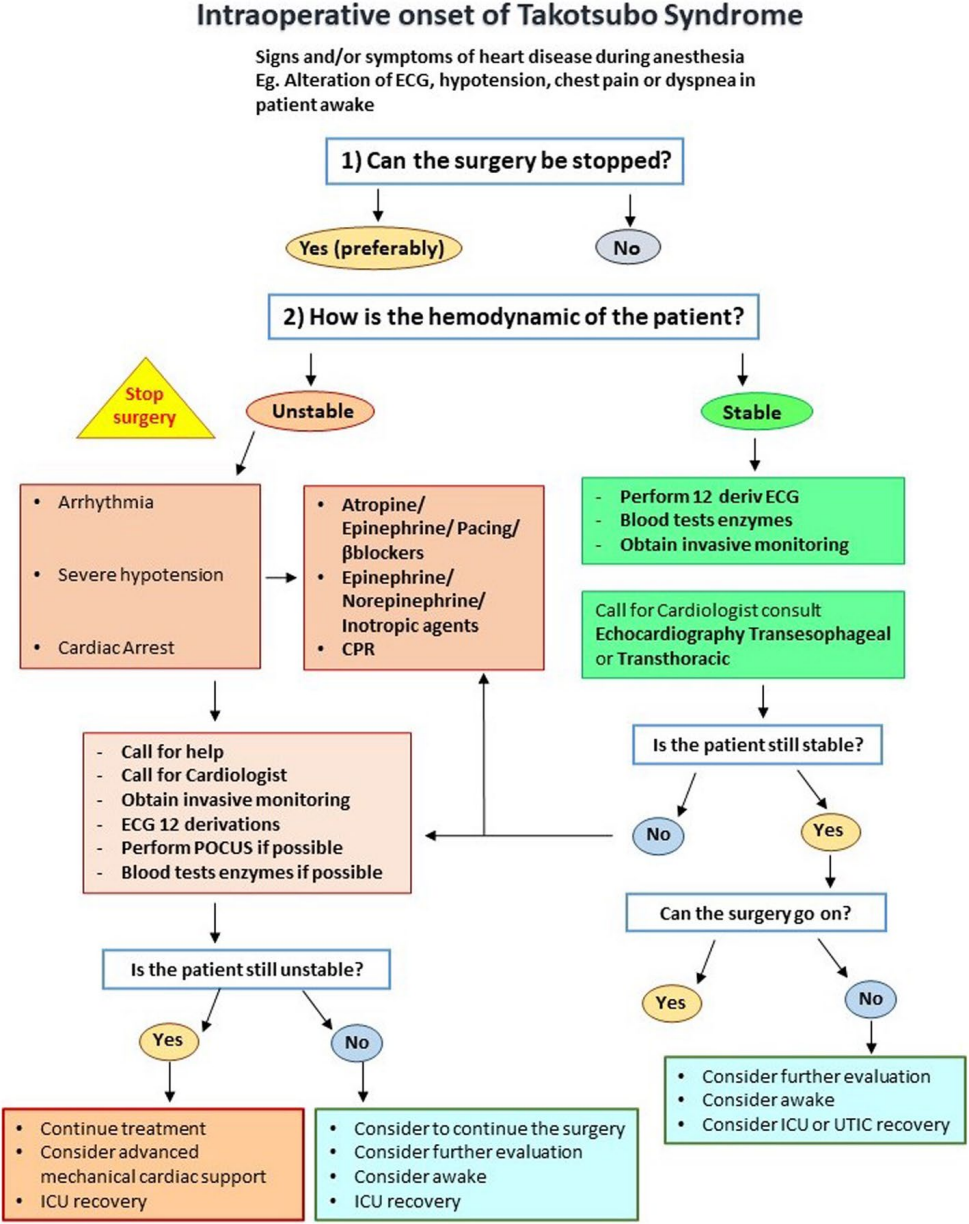
Flowchart of TTS management

**Fig. 6:**
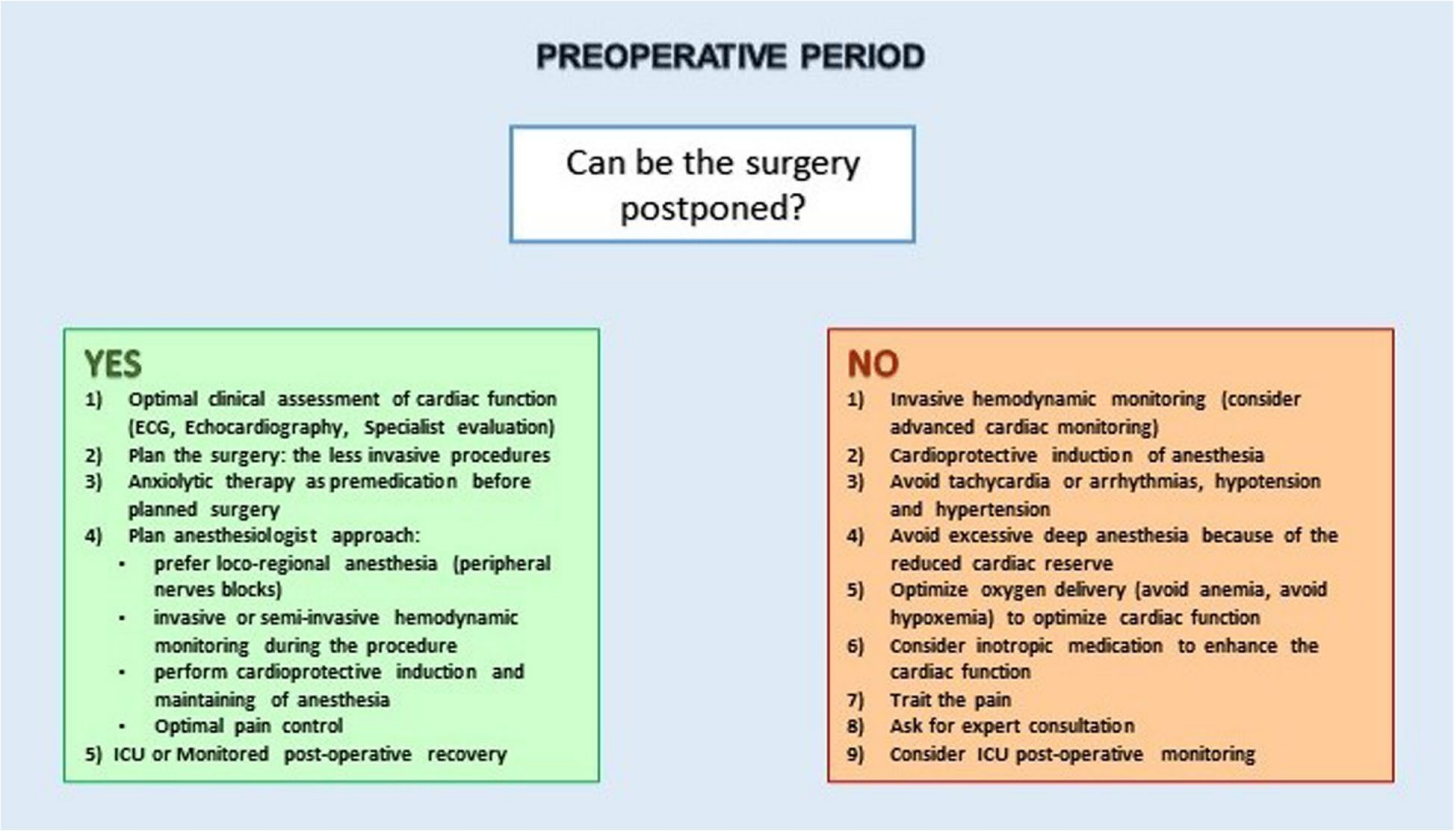
Treatment strategy based on the type of surgery; elective vs emergent

The current existing literature on the management of Takotsubo during the perioperative period is limited, and no universally accepted guidelines are currently available. Consequently, the treatment of TTS relies on health care personal experience and/or local practice. In the perioperative setting, an anaesthesiology can be called to face several possible scenarios: the management of a patient with the diagnosis of new-onset Takotsubo before elective surgery, the need to manage an emergent surgery in a patient with a concomitant stress cardiomyopathy and stress cardiomyopathy arising as a complication during surgery. A flowchart of possible TTS management is presented in Fig. 5. The first aspect that an anaesthesiologist must evaluate is the kind of surgery, elective versus emergent (as shown in Fig. 6). Given the reduced cardiac reserve, elective surgery must be postponed in stress cardiomyopathy [59, 60]. Some authors suggest postponing elective interventions if potential psychological/emotional triggers such as recent major bereavements emerge during the preoperative assessment [61]. When deferral is not an option, some considerations can assist in the management. Preoperative evaluation includes the diagnostic workup, typical of the cardiological evaluation and should focus on the severity of the condition. The first-line assessment is characterized by the ECG, transthoracic echocardiography (TTE) and cardiac enzymes, especially for the differential diagnosis with acute coronary syndromes. Subsequently, coronary angiography and transoesophageal echocardiography (TEE) should also be considered. In this setting, cardiological consultancy is fundamental for the best standard of care. In addition, it is important to perform chest radiography or lung ultrasonography to rule out possible complications (e.g. pulmonary oedema). Blood sample should also be collected for the evaluation of electrolytes, liver function, renal function and coagulation profile.

When the acute phase is over, elective surgery should be postponed until patients with TTS are in the recovery phase and have met the criteria of stability assessed by specialist consultation. Then, on the day of surgery, regardless of the anaesthetic technique chosen, it is important to limit stressful situations that could lead to an increase in circulating catecholamine levels. And it is also important to consider a good preoperative anxiolysis by combining the psychological approach with the pharmacological one [62]. Regional anaesthesia, alone or in combination with general anaesthesia (i.e. blended anaesthesia), should always be considered in the light of its effect on the autonomic system as well as its role in providing optimal control for both intraoperative and postoperative pain. These effects can represent vital aspect able to spare the stress associated with tracheal intubation and extubation [63]. However, published case reports describe the onset of stress cardiomyopathy immediately after the spinal anaesthesia; consequently, it can be speculated that not all loco-regional anaesthesia techniques can be considered “stress-free” [64, 65]. Compared to neuraxial technique which may potentially have a greater haemodynamic impact, peripheral nerve blocks appear to be safer, when appropriate for the type of intervention, in patients with a history of stress-induced cardiomyopathy [63]. However, the effects of epidural anaesthesia on reducing pain and on limiting the autonomic system activation can be very favourable in this context [66]. Logically, a post operative ICU stay must be considered.

When it is not possible to postpone surgery (i.e. urgent or emergent cases), particular considerations have to be taken into account. Induction of general anaesthesia should aim to preserve cardiac function with cardioprotective objective, favouring a multimodal approach. Anaesthetics have to be chosen in order to minimize myocardial depression and to guarantee an excellent analgesic plan with the objective to reduce stress and the consequent release of catecholamines [67]. The anaesthesia depth is essential, but it is also important limiting the anaesthetic doses due to the reduced myocardial reserve [68]. Even more, maintenance of sinus rhythm and avoidance of tachycardia or bradycardia are essential. Together with standard monitoring, invasive monitoring has also to be utilized. An arterial line for evaluation of the invasive blood pressure represents a cornerstone of the haemodynamic evaluation [69, 70]. However, the current variety of haemodynamic monitoring tools allows the clinician to have more advanced haemodynamic parameters, such as cardiac output and stroke volume variation. In this respect, there are currently several minimally invasive options, like cardiac output monitoring devices based on the pulse contour analysis, such as PICCO (Getinge Ltd), Most Care (Vygon Ltd) and LiDCO (Masimo Ltd) [71, 72]. Alongside the cardiac function, the advanced monitoring can provide important information regarding the amount of extravascular lung water (EVLW) [73]. These data are crucial not only for the rapid identification of possible intraoperative complication but also for a correct administration of fluid [74]. For the same reason, strict control of the urinary output must be obtained. Intraoperative TEE can also be considered; however, this technology is not widely available in non-cardiac operating rooms. It is also pivotal to insert a central line catheter for the administration of vasoactive drugs. Finally, particular attention must be given to the patients’ position on the operative table due to the fact that even minimal variations of the patient’s posture can have a huge impact on the haemodynamic status.

When stress cardiomyopathy occurs during general anaesthesia, other potential causes of acute onset of hemodynamic instability should always be considered: all the tools described above can help to guide the diagnosis.

In these cases, the intervention is generally interrupted and postponed until of cardiac function has fully recovered [75]. Sometimes, stress cardiomyopathy can occur in patients without apparent risk factors, which makes the diagnosis more difficult, if this form is not considered early in the differential diagnosis [76]. In these cases, no advanced hemodynamic monitoring may have been planned, especially in the case of non-major surgery. Therefore, the only clues can be the acute electrocardiographic changes and hypotension detected by non-invasive blood pressure monitoring [76]. In these circumstances, a more advanced monitoring should be implemented if possible, and urgent cardiologic consultation should be requested. In all cases of onset of stress cardiomyopathy in the perioperative period, adequate monitoring and treatment should be guaranteed in intensive care, given the high risk of persistent haemodynamic instability and further complications[77].

## Treatment and postoperative care

There is no shared optimal pharmacological treatment for stress cardiomyopathy, as this greatly depends on the severity of the clinical picture and the onset of any complications. Certainly, as in all phases of the disease, in the postoperative management, it is also essential to contain risk factors, by offering good pain control and by limiting other stressors, including respiratory fatigue [78].

Ideally, patients with cardiogenic shock should be managed in tertiary care centres, with easier access to advanced mechanical cardiac support devices (i.e. ECMO, IABP and LVAD), particularly in cases of shock refractory to medical therapy [79]. The utilization of mechanical cardiocirculatory support (MCS) in TTS has increased in the last few years. However, up to now, the paucity of literature on the use of MCS in TTS patients makes it hard to come to any kind of evidence-based recommendations about the efficacy and the safety of the different devices (VA-ECMO, IABP and LVAD). The timing of implantation and weaning is also not clear in this population. In 2024, Von Mackensen et al. published a systematic review on the use of MCS for TTS patients [80]. The authors included 93 articles from 124 patients; 50% were treated with veno-arterial extracorporeal membrane oxygenation (VA-ECMO), 36% received a left ventricular assist device (e.i. Impella) and 10% IABP. The initial left ventricular ejection fraction was 20% for VA-ECMO and Impella, whereas the left ventricular ejection fraction was around 30% for IABP group. The survival rate was 87.1% for VA-ECMO, 84.1% for Impella and 84.6% for IABP. Full recovery was observed in 75.8% for VA-ECMO, 72.7% for Impella and 61.5% for IABP. However, due to the heterogeneity of the population included, the authors concluded that the data are insufficient to draw any conclusion regarding the superiority of one treatment over the others. Mariani et al. published in 2020 a systematic review and meta-analysis on the use of MCS in TTS patients [81]. The authors included 81 studies and 93 MCS cases with a survival rate of 94.6%. The median left ventricular ejection fraction was 20% before the implantation, with a median time on MCS of 3 days. The time on MCS, which is relatively short compared to other cases with different aetiology of cardiogenic shock, could reflect the transient cardiac function impairment typical of TTS [81]. The authors also observed that the use of IABP was common before 2012, while VA-ECMO or LVAD, especially Impella, was more frequent in the recent years, with VA-ECMO implantation more utilized in younger patients. Blood pressure, left ventricular ejection fraction, heart rate or TTS type did not seem to affect the choice of the device. However, it is important to consider some particular aspects: is in the case of LVOTO, for instance, the IABP can worsen the haemodynamic status and should be avoided. The authors also suggested an early application of MCS in TTS to reduce or prevent the excessive use of exogenous catecholamines, which could worsen the pathology, and to prevent refractory shock or cardiac arrest. Conversely, rescue or late MCS implantation delays hemodynamic support, which impairs the efficacy of the therapy and requires emergency insertion, which clearly may increase potential complications. A multicentre retrospective analysis on the use of Impella for TTS with cardiogenic shock was published in 2021, describing 16 cases with similar left ventricular ejection fraction (20%). The Impella device was in place between 1 and 4 days [82]. Eighty-one percent of the patients survived to discharge with complete recovery of cardiac function and with improvement of LVEF at discharge compared to the baseline [82]. These results are encouraging; however, further studies are needed on this topic.

In terms of pharmacological treatment, the drugs commonly used for heart failure include ACE inhibitors, β-blockers and diuretics; these are cornerstones of the management of myocardial dysfunction in stress cardiomyopathy [83]. In particular, β-blockers have a role in reducing the extent of LVOT obstruction [84]. The rationale for their use can be understood in relation to the role that alpha and β-adrenergic stimulation has in the pathogenesis of stress cardiomyopathy; they exert their clinical effect by reducing the contractility at the base of the heart and by facilitating the diastolic filling of the left ventricle. Both these effects contribute to reduce the gradient through the left ventricular outflow tract and its deleterious consequences [85]. Particularly, the use of the β-selective β-blocker esmolol has been proposed in the acute phase, given its extremely short half-life that makes it an easily titratable agent [86]. However, β-blockers remain contraindicated in cases of acute heart failure with low left ventricular ejection fraction, hypotension and bradycardia; those are cases in which the negative chronotropic and inotropic effects of β-blockers could be deleterious [44]. Furthermore, the long-term prognostic value of β-blocker remains controversial as it would appear that their use does not prevent the recurrence of stress cardiomyopathy [87], as also reported by the International Expert Consensus Document on Takotsubo Syndrome [44]. Diuretics play a role in reducing dyspnoea associated with LVOTO; however, they must be carefully administered to avoid induction of hypovolemic states [49].

The case of stress cardiomyopathy complicated by cardiogenic shock presents an additional challenge. Indeed, while it would normally seem appropriate to use inotropes, they should be avoided as they further maintain and stimulate the pathophysiological mechanisms underlying stress cardiomyopathy [52]. More specifically, catecholamines such as adrenaline, dopamine and dobutamine, as well as the phosphodiesterase inhibitor milrinone all increase the oxygen consumption. Levosimendan, on the other hand, is a non-catecholaminergic inotrope, which improves the myocardial contractility without an increase in calcium influx across the cardiomyocyte membrane, unlike the above-mentioned agents. Levosimendan prolongs the interaction between actin and myosin filaments during systole, when intracellular calcium levels are at their highest, thus increasing myocardial systolic performance and therefore cardiac output. It also acts by opening ATP-dependent potassium channels, causing both systemic and pulmonary vasodilation [88]. Therefore, when inotropic support is needed, the drug of choice would appear to be levosimendan [53]. There is also evidence in favour of milrinone, though limited to some case reports [89]. Among catecholamines, noradrenaline deserves a separate discussion. In fact, given its stimulating effects of both alpha and β adrenergic, noradrenaline is probably safer than other catecholamines. This is because, in addition to the positive inotropic activity, it possesses vasoconstrictive activity on both the arterial and venous districts, increasing, in this way, the preload and afterload. However, cases of precipitated LVOTO after administration of noradrenaline to counteract vasoplegia have been reported [90]. As already illustrated, volume filling also plays a role in the mechanisms that lead to LVOTO. In hypovolemic patients, prudent administration of fluids during LVOTO improves preload and ventricular filling status; however, this should be guided by adequate hemodynamic monitoring, as already described [47].

## Outcome of TTS in the perioperative settings

Given the limited series in specific settings, data on the prognosis of perioperative stress cardiomyopathy are extrapolated from studies which also include non-surgical patients. Although previously described as a benign syndrome, due to the relatively rapid restoration of normal cardiac function in most cases, an increased number of study studies have confirmed that some cardiac dysfunction may persist despite the normal ejection fraction in many patients [10]. Furthermore, these patients have a morbidity and mortality rate that is largely comparable to that of patients with classic acute coronary syndromes [91].

The growing availability of literature on the topic has identified a few parameters as independent risk factors for a worse prognosis: age, a left ventricular ejection fraction, higher troponin values, the presence of a physical trigger, atypical patterns, cardiogenic shock or cardiac arrest [92]. The International Takotsubo Registry, including 1750 patients of 26 European and United States centres, reported a mortality of 5.6% per patient-year and a rate of major adverse cardiovascular events of 9.9% patient/year [30]. Interestingly, in comparison with ACS, neurologic or psychiatric complications were higher (55.8% vs. 25.7%, *p* < 0.001) and the mean left ventricular ejection fraction was lower (40.7 ± 11.2% vs. 51.5 ± 12.3%, *p* < 0.001). In 2019, a systematic review and meta-analysis by Pelliccia et al., including 54 studies with a total of 4679 patients, reported a more benign long-term survival with an annual rate of total mortality of 3.5%. Meta-regression analysis found that mortality was significantly associated with older age (*p* = 0.05), physical stressor (*p* = 0.0001) and the atypical ballooning form (*p* = 0.009) [93].

## Special setting and population

### Ambulatory and non-operating room anaesthesia

Ambulatory and non-operating room anaesthesia (NORA) represent a growing field of the anaesthesiologist’s practice. Often performed in remote location of the hospital, must face particular challenges both in term of limited access of anaesthesia equipment and lack of support of trained staff. Even more, the unfamiliarity of the settings represents a further risk. Even if the procedures performed in this environment are generally less invasive in comparison with the ones performed in the operating room, the anaesthesiologist must be aware that Takotsubo cardiomyopathy may also affect these low-risk patients.

Several case reports are published on the occurrence of Takotsubo during gastrointestinal endoscopies [94–97]. Even more, Brunetti et al. reported a case of “ultra-fast Takotsubo cardiomyopathy” during eye surgery [98]. Interestingly, the LV dysfunction recovered in one day and the authors highlight the possibilities that the incidence of TTS in the clinical practice could be even higher than actually estimated.

### OB/GYN

Takotsubo cardiomyopathy is usually associated with postmenopausal women. However, several case reports are published on pregnancy-associated TTS [99–102]. Therefore, the anaesthesiologist should be aware of the potential of this severe complication in the challenging peripartum phase. The clinical manifestation is characterized by chest or epigastric pain, mimicking acute cardiac ischaemia.

Minatoguchi et al. reviewed 18 cases of peripartum TTS, which occurred generally after caesarean delivery (81%) [103]. Remarkably, the symptoms were undistinguishable from ACS (e.g. chest pain in 44% and dyspnoea in 28% of the cases) with ECG abnormalities in 94% of the cases (e.g. both ST changes and T-wave inversion). Papadopoulos et al. in 2021 tried to investigate the catecholamines stress during vaginal and caesarean delivery to explain the difference in the incidence of TTS, quoting the autonomic imbalance as the potential cause [104]. They found lower noradrenaline blood levels and vagal withdrawal in women undergoing caesarean delivery in comparison to women who delivered naturally; they attributed this autonomic imbalance to the difference in the drugs’ administration between these two types of labour, postulating a protective role of the oxytocin infusion and considering general anaesthesia and neuraxial anaesthesia as precipitating factors for the cardiomyopathy.

TTS can also occur during preeclampsia or HELLP syndrome [105–107]. Oindi et al. described a case report of stress-induced cardiomyopathy in a woman with severe preeclampsia at 31 weeks of gestation. The authors stated that a depressive trauma due to a prior unfavourable pregnancy, in addition to the preeclamptic status may have represented the possible trigger for TTS. Fortunately, cardiac function fully recovered with no major adverse effects on the new-born baby. Gabarre et al. described another case report of inverted Takotsubo syndrome in a patient with HELLP syndrome [106]. In this case, the woman had an uncomplicated spontaneous vaginal delivery, but she was given extremely distressing news about her child just after delivery: she soon showed signs of inverted Takotsubo syndrome. Finally, another interesting case report was described by Kraft et al. Takotsubo syndrome occurred after an emergency caesarean section due to foetal bradycardia and breech presentation; during the operation, the patient had pulseless ventricular tachycardia very quickly self-terminating; however, few hours later, the woman’s clinical condition worsened, with signs of acute heart failure. The ECG showed prolonged QTc but no ST elevation, the blood samples showed a moderate increase of troponin T concentration and the echocardiogram showed a significant reduction of the ventricular ejection fraction with the typical apical ballooning. Luckily, the patient’s clinical conditions improved rapidly. After approximately 4 days, the patient recovered completely without sequelae [107].

## Conclusions

The increasing awareness about TTS, not only as a consequence of some emotional stress, either positive or negative, but also in the setting of the perioperative period and/or critical illnesses, demands a more vigilant approach to this population of patients. In most of the cases of TTS, the short-term prognosis is favourable, with a complete recovery of the regional and global ventricular function at the hospital discharge; however, serious complications can occur, with approximately 20% of patients evolving to a full-blown cardiogenic shock with the potential need for advanced support treatment and increased mortality.

The approach to a patient who develops the syndrome in the perioperative period should include delaying surgery, allowing clinicians to optimize the cardiac function before the surgical stress. If delaying is not possible, a careful anaesthesiologic plan must be put in place. It is important to remember that TTS can also develop in cases of less invasive procedures, such as ambulatory surgery and NORA settings. Moreover, the peripartum period, especially if the caesarean section is needed, can expose the patients to the risk of TTS. It is fundamental to diagnose TTS as soon as possible and choose the best supportive care. Due to the heterogeneity of the presentation and the potential severity, a deep knowledge of the syndrome and related management is warranted, with the aim of reducing mortality and morbidity of these patients.

### Supplementary Information

Additional file 1: Supplemental material 1. 3D ejection fraction display of a typical form of TTS: the apical dyskinesia and ballooning is clearly shown.

## Data Availability

No datasets were generated or analysed during the current study.

## Abbreviations

TTS: Takotsubo syndrome (TTS)
ECG: Electrocardiogram
PKA: Protein kinase A
ATP: Adenosine triphosphate
STEMI: ST-Segment elevation myocardial infarction
N-STEMI: Non ST-segment elevation myocardial infarction
CK: Creatine kinase
CK-MB: Creatine phosphokinase-MB
Intertak: International Takotsubo
LVOTO: Left ventricular outflow tract obstruction
TTE: Transthoracic echocardiography
ICU: Intensive care unit
PICCO: Pulse Index Continuous Cardiac Output
EVLW: Extravascular lung water
ACS: Acute coronary syndrome
ECMO: Extra corporeal membrane oxygenation
IABP: Intra-aortic balloon pump
LVAD: Ventricular assist device
ECMO V-A: Artero-venous extra corporeal membrane oxygenation
MCS: Mechanical circulatory support
ECPR: Extracorporeal cardiopulmonary resuscitation
ACE inhibitors: Angiotensin-converting-enzyme inhibitors
AMP: Adenosine monophosphate
NORA: Non-operating room anaesthesia
EGDS: Esophagogastroduodenoscopy
HELLP syndrome: Hemolysis, elevated liver enzymes, and low platelet count
OB/Gyn: Obstetrics/gynaecological
